# Understanding hope at diagnosis: A study among Guatemalan parents of children with cancer

**DOI:** 10.1002/cam4.5725

**Published:** 2023-02-27

**Authors:** Anneliese H. Williams, Silvia Rivas, Lucia Fuentes, Ana Cáceres‐Serrano, Gia Ferrara, Tegan Reeves, Federico Antillon‐Klussmann, Carlos Rodriguez‐Galindo, Jennifer W. Mack, Dylan E. Graetz

**Affiliations:** ^1^ Purdue University West Lafayette Indiana USA; ^2^ Unidad Nacional de Oncología Pediátrica Guatemala City Guatemala; ^3^ St. Jude Children's Research Hospital Memphis Tennessee USA; ^4^ Francisco Marroquin University School of Medicine Guatemala City Guatemala; ^5^ Dana Farber Cancer Institute/Boston Children's Hospital Boston Massachusetts USA

**Keywords:** clinical cancer research, pediatric cancer, prognosis, psychosocial studies

## Abstract

**Background:**

In high‐income countries, hope facilitates parental coping and builds the clinical relationship between families of children with cancer and their clinicians. However, the manifestation of hope in low‐ and middle‐income countries (LMICs) remains poorly understood. Our study explores Guatemalan parents' experiences with hope during the pediatric oncology diagnostic process and aims to identify discrete actions clinicians take to support hope.

**Methods:**

This qualitative study utilized audio‐recordings of the diagnostic process and an additional semi‐structured interview for 20 families of children with cancer at Unidad Nacional de Oncología Pediátrica in Guatemala. Spanish audio‐recordings were translated into English, transcribed, and coded using a priori and novel codes. Thematic content analysis using constant comparative methods explored parents' hopes and concerns.

**Results:**

At diagnosis, Guatemalan parents expressed both hopes and concerns related to the entire cancer continuum. Throughout the diagnostic process, hope grew as concerns were alleviated. Clinicians supported hope by creating a supportive environment, providing information, affirming religious beliefs, and empowering parents. These strategies helped parents shift their focus from fear and uncertainty toward hope for their child's future. Parents expressed that establishing hope improved mood, promoted acceptance, and enabled them to care for themselves and their children.

**Conclusion:**

These results confirm the relevance of supporting hope in pediatric oncology settings in LMICs and suggest that culture informs hope‐related needs. Supporting hope is critical across cultures and can be integrated into clinical conversation using the four processes identified by our results.

## Graphical abstract

This study describes four actions clinicians can use to support hope across culture and resource setting, which ultimately improve parental outcomes and facilitate clinical relationships.

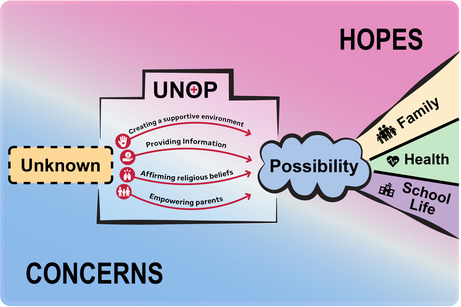

## 1 INTRODUCTION

For parents of children with cancer, hope has been demonstrated to alleviate distress and support a positive outlook.^1^ Hope‐centered conversations help clinicians foster trust and build the clinician–family relationship.^2^, ^3^ However, the majority of hope research has been conducted in high‐income countries (HICs) and in the setting of poor prognosis. In these contexts, parents emphasize the importance of hope, describing it as a guiding force that gives them the strength to navigate the uncertainties of cancer treatment.^1^, ^4^ Additionally, parental hope fluctuates as their child's condition changes and parents' ability to focus on the positive is challenged by factors like uncertainty and fear.^5^ How parents of children with diverse prognoses balance hope and concern from the time of diagnosis remains poorly understood, particularly in low‐ and middle‐income countries (LMICs), where 90% of children with cancer live.^6^

Hope theory suggests a goal‐pathway‐agency model. According to this model, to have hope an individual must be able to identify what they want to happen (a goal), believe a path to their desired outcome exists (a pathway), and feel capable of following that pathway (agency).^7^, ^8^ Hopelessness arises when an individual feels unable to change their circumstances in lieu of their goals.^8^ A recent extension of hope theory suggests that hope has a culture‐dependent “locus” dimension.^7^ In individualist cultures, hope relies on cognitions about one's own ability to influence outcomes (an internal locus), whereas in collectivist cultures people anchor hope externally, deriving hope from beliefs about how family, friends, and supernatural beings can influence outcomes (an external locus).^7^ These findings highlight the need to extend research on hope in pediatric oncology beyond HICs to settings with different customs and beliefs. The purpose of this analysis was to explore hopes and concerns of parents of children with cancer, including a range of prognoses, during diagnostic conversations in Guatemala, a collectivist,^9^ upper‐middle income country.^10^

## 2 METHODS

This is a secondary analysis of a study for which complete methodology has been previously reported.^11^ Details pertaining to this analysis are described below.

### 2.1 Settings and participants

This study was conducted at Unidad Nacional de Oncología Pediátrica (UNOP) in Guatemala, which is funded through a public‐private partnership and is Guatemala's only national pediatric cancer center. Guatemala is a small diverse country with 24 principal ethnic groups and a collectivist culture in which identity is centered in the family or community group rather than in individual traits.^9^ UNOP uses a multidisciplinary approach known as “medicina integral” which provides psychosocial services and resources to help cover expenses for families with children in treatment. The childhood cancer survival rate in Guatemala is ~65%.^12^

Spanish‐speaking parents of children under age 18 with a new or probable cancer diagnosis were eligible to participate in this study. Families were approached sequentially with additional purposive sampling to ensure a range of ages, diagnoses, and socioeconomic statuses. Data was collected between April and August of 2019; 32 families were approached, 20 chose to participate. Most families who declined participation cited hesitancy about being audio‐recorded; one family was unable to participate due to language. The children of families who declined participation had similar diagnoses, ages, and genders to those of participating families. Participants gave written informed consent prior to participation. Study design complied with international regulations for the protection of human subjects and was approved by the UNOP ethics committee and St. Jude IRB.

### 2.2 Study design and data collection

At UNOP, parents participate in a two‐part diagnostic process. Initially, parents meet with a psychologist who conducts a psychosocial intake and provides a general explanation of cancer. Within approximately a week, an oncologist provides the official diagnosis and treatment plan with the psychologist present for support. In this study, both conversations were audio‐recorded, and a recorded semi‐structured interview of one parent was conducted within 14 days of diagnosis. In interviews, parents expanded on experiences with the diagnostic process and reflected on hopes and concerns.

All study materials including the protocol and interview guides were translated and reviewed by bilingual team members. The interview guide was iteratively revised in Spanish and back translated into English. Audio‐recordings of conversations and interviews were professionally transcribed and translated into English; bilingual research team members checked translated texts against Spanish recordings to verify the transcripts accurately reflected the content of original audio recordings.

### 2.3 Data analysis

Analysis was conducted using a priori and novel codes. Two authors coded each transcript independently and disagreements were resolved by consensus with a third‐party adjudicator. All data was coded for whether a parent, psychologist, or oncologist was speaking. This secondary analysis focused on data coded under a priori code “Expression of concern”^13^ defined as, “Utterances in which the caregiver expresses worry, anxiety, fear, anger, frustration, and other forms of negative affect or emotion; includes references to things being hard or difficult” and novel code “Expression of hope”, defined as “Utterances in which the caregiver expresses hope, desire for the future, or other form of positive emotion”. Additional novel codes informed the analysis including, “Uncertainty”, “God/Faith/Fatalism”, “Family Factors”, and “Child's Best Interest”.^11^ Thematic content analysis using constant comparative methodology^14^, ^15^ explored parental perceptions of hope and its perceived impacts during the diagnostic process. MAXQDA software facilitated data management.^16^ The Consolidated Criteria for Reporting Qualitative Studies guidelines were used to ensure rigor.^17^

## 3 RESULTS

### 3.1 Demographics

Most families were Christian; 65% identified as Evangelical and 20% identified as Catholic. Although 80% of patients had a hematological malignancy, a range of other diagnoses were represented. Participating clinicians included three psychologists and seven pediatric hematologist/oncologists. Complete family and clinician demographic information is included in Table 1.

**TABLE 1:** Participant demographics *n* (%).

Family demographics	
Patient demographics	
Patient age	
<4.9 years	6 (30)
5–9.9 years	6 (30)
10–14.9 years	4 (20)
15–18 years	4 (20)
Patient gender	
Male	11 (55)
Female	9 (45)
Diagnosis	
Hematological malignancy	16 (80)
Solid tumor	3 (15)
CNS tumor	1 (5)
Parent demographics	
Religion	
Evangelical	13 (65)
Catholic	4 (20)
Other	3 (15)
Primary language	
Spanish	12 (60)
Mayan dialect (Kaqchikel, Kekchi, Mam, Quiche, Pocomam)	8 (40)
Number of children	
1	2 (10)
2–3	7 (35)
4–5	6 (30)
>6	5 (25)
Distance to UNOP	
<2.5 h	4 (20)
2.5–5 h	3 (15)
>5 h	6 (30)
Family home	
Rural	18 (90)
Urban	2 (10)
Mother's employment	
Housewife	18 (90)
Dayworker	1 (5)
Professional	1 (5)
Father's employment	
Unemployed	1 (5)
Dayworker	1 (5)
Professional	8 (40)
Farmer	9 (45)
Unknown	1 (5)
Mother's education level	
None	6 (30)
Elementary	8 (40)
Beyond elementary	1 (5)
Unknown	5 (25)
Father's education level	
None	2 (10)
Elementary	15 (75)
Beyond elementary	3 (15)

Clinician demographics	
Discipline	
Pediatric hematologist/oncologist	7 (70)
Psychologist	3 (30)
Age	
20–29 years	2 (20)
30–39 years	3 (30)
40–49 years	2 (20)
50–59 years	3 (30)
Sex	
Female	6 (60)
Male	4 (40)
Years of experience	
1–3 years	4 (40)
4–6 years	2 (20)
7–9 years	1 (10)
10+ years	3 (30)

Participation information	
Parent present for psychosocial intake	
Mother	9 (45)
Father	5 (25)
Both	6 (30)
Parent present for diagnosis with oncologist	
Mother	7 (35)
Father	5 (25)
Both	8 (40)
Parent interviewed	
Mother	13 (65)
Father	7 (35)

### 3.2 Hopes and concerns across the cancer continuum

At diagnosis, parents expressed a breadth of hopes and concerns that pertained to the entire cancer continuum.

Parents reported a “*pre‐diagnosis*” process prior to arrival at UNOP, citing expensive tests, unsuccessful treatments, and worsening symptoms. During this time, lack of knowledge about why their child was sick or what to do created helplessness and fear. One parent said, “Because we didn't know what to do with her back there… right now I'm with the anxiety to know what's wrong with her, the nurse said we don't know, and I asked why, why” (mother of a child with a hematological malignancy, psychosocial intake). Concerns dominated parents' reflections on the pre‐diagnosis period, although some expressed hope that UNOP would be able to treat their child.

During the transition to UNOP, hopes and concerns about “*diagnosis*” arose. Parents worried about the cause of cancer, prognosis, and extent of disease. One parent explained, “My family and I were really worried because we thought cancer didn't have a cure” (father of a teenager with a hematological malignancy, interview). Some parents expressed hopes for a favorable diagnosis, good prognosis, and treatment options: “If the diagnosis is positive, I trust the doctors here, that they will give him the right treatment” (mother of a child with a hematological malignancy, psychosocial intake).

Once parents met with clinicians at UNOP and heard a cure might be possible, they articulated hopes and concerns related to “*treatment*.” Guatemalan parents worried about disease progression, interventions, and acute toxicities. A mother relayed her concern, “he is not eating enough, because the doctor said that if he doesn't eat, he can be malnourished, and he can even require a tube” (mother of a child with a hematological malignancy, interview). Parents hoped for positive disease response and minimal treatment side effects. Many participants hoped to return home. One parent shared her hope “that my girl can leave before the two years of treatment,” (mother of a child with a hematological malignancy, interview).

Parents also considered life “*after completion of therapy*,” seeking normality for their child, including graduation, marriage, and long‐term health, from the time of diagnosis. One Guatemalan parent hoped that her child, “… could grow, play, and have a happy life” (mother of a child with a hematological malignancy, interview). Thoughts about life after treatment were predominantly hope‐focused; however, participants also expressed concerns about bullying, long‐term side effects, and relapse.

Table 2 provides additional quotes describing hopes and concerns related to each phase of the cancer continuum.

**TABLE 2:** Hopes and concerns across the cancer continuum anticipated at diagnosis.

	Concerns	Hopes
Reflections on the *pre‐diagnosis* period	“I was worried because of her tachycardia, blood pressure, a lot of cough, and she was bruised and vomiting.” (mother of a teenager with a hematological malignancy, interview) “We are stranger people coming from far away, and we did not know if you were going to take us, we thought maybe discrimination, because we are not like people here.” (father of a child with a hematological malignancy, psychosocial intake)	“We were hoping that it was a simple sickness.” (father of a child with a hematological malignancy, interview)
Anticipations about the implications of the *diagnosis*	“When I first went there, he had a little ball and now it's so big…and then another one, he's got another one on his stomach, and that's why I'm so scared.” (father of a child with a hematological malignancy, psychosocial intake) “My doubts were…watching children here without arms of legs, without eyes or hair…I was very worried.” (mother of a child with a hematological malignancy, interview)	“But when my family comes to see me I do explain, I tell them that ALL is cancer and it sounds harsh but there is hope. I trust God and the foundation that she is going to be fine.” (mother of a teenager with a hematological malignancy, interview)
Anticipations about what *treatment* will entail	“I felt sad with some questions, I mean, she is girl and has long hair. I asked if she was going to lose her hair and they said yes that all the children had to deal with that.” (mother of a child with a hematological malignancy, interview) “But it was hard, he would vomit every meal, he had chemo and lost a lot of weight.” (mother of a child with a hematological malignancy, interview) “I feel worried with this disease; I'm always thinking if it can progress and spread or if this is going to be cured.” (mother of a child with a hematological malignancy, interview)	“I already know the illness and I'm going to pray to God can give her strength, I hope she'll respond well to the treatment.” (father of a teenager with a hematological malignancy, diagnostic conversation) “What I want is to watch her standing. What her to be cured.” (mother of a child with a hematological malignancy, interview) “That's what I hope for, no surgery.” (mother of a child with a hematological malignancy, psychosocial intake)
Anticipations about how *life after completion of therapy* will be affected	“And other children will call her names.” (mother of a child with a hematological malignancy, diagnostic conversation) “No, in the sense that I'm worried he could relapse.” (mother of a child with a hematological malignancy, interview) “I'd like to ask one question, you know she's a girl and she's getting to the stage of women's development, and I wanted to ask if that will be affected.” (mother of a child with a hematological malignancy, diagnostic conversation)	“Yes. I hope that my son can study, God's will he will continue his studies.” (mother of a teenager with a solid tumor, interview) “That my baby can get married” (father of a child with a hematological malignancy, diagnostic conversation) “I know I'm going to take him home; he'll watch tv and football games, he'll play” (mother of a teenager with a solid tumor, psychosocial intake)

### 3.3 Supporting hope

The clinical team at UNOP facilitated hope in four discrete ways: *creating a supportive environment*, *providing information*, *affirming religious beliefs*, and *empowering parents*.

Staff at UNOP *created a supportive environment* that encouraged hopes and mitigated concerns. Parents' concerns were amplified by separation from support systems. UNOP staff reassured parents that they were not alone. One participant voiced the importance of this explaining, “Sometimes the support we need is not monetary but morally, they encourage us to keep fighting” (mother of a teenager with a hematological malignancy, interview). Clinicians established shared priorities to build a supportive environment, “We always ask this because whatever is important to you, it's important to us” (psychologist to parents of a child with a hematological malignancy). Parents found these interactions helpful for maintaining a positive outlook. One explained, “The staff, the workers, maintenance, the nurses, the doctors sometimes come inside and chat a little and say don't worry you will be home soon. It's a hope” (father of a child with a hematological malignancy, interview).

Clinicians also *provided information* which supported hope. During diagnostic conversations, clinicians focused on cure and addressed fatalistic beliefs. One oncologist explained, “there is a possibility and that's what we want to offer you today, treatment” (oncologist to parents of a teenager with a hematological malignancy). Parents acknowledged the impact of information. One parent said, “Now that you talked to us, we have hope and now we understand more about the illness,” (father of a child with a hematological malignancy, diagnostic conversation).

Clinicians *affirmed religious beliefs* during diagnostic conversations by establishing shared values, encouraging coping strategies like prayer, and highlighting God's role. One psychologist explained, “Just like you, we are people of faith here at the hospital” (psychologist to a father of a teenager with a hematological malignancy). Clinicians emphasized the possibility of a cure using religious language, “Sometimes we ask God for a miracle, and he uses humans to do it. And he left knowledge to doctors” (psychologist to parents of a child with a hematological malignancy) to reinforce faith in treatment and establish a connection between the clinicians and God.

Finally, the UNOP team *empowered parents*. Participants expressed distress related to feeling unable to help their child; one told a psychologist, “I'd like to do something, but I cannot do anything” (father of a child with a hematological malignancy, psychosocial intake). In response, clinicians emphasized needs parents could fulfill: “give her love, letting her know she's not alone, that's what you can control” (psychologist to a father of a teenager with a CNS tumor). Clinicians also articulated ways parents could facilitate treatment. They encouraged parents to find blood donors and emphasized the importance of bringing the child to appointments. One psychologist explained, “Bring him to his appointments and as [the] hospital, we will give him his treatment, and God will do his part as well” (psychologist to a father of a child with a hematological malignancy).

As clinicians created a supportive environment, provided information, affirmed religious beliefs, and empowered parents, they strengthened trust between the healthcare team and families, establishing a pathway for parents to embrace hope. One parent described this impact saying, “Well, yes, my vision changed because it was told to me by a doctor, a person with experience, he gave me more hope” (father of a teenager with a hematological malignancy, interview). In addition to the environment of trust and support cultivated by clinicians, some parents described how their religious beliefs and community or family support facilitated a growing sense of hope: “Even though it was hard for me to accept it, my husband told me to have faith that we will be in those 7 kids. There is the hope” (mother of a child with a hematological malignancy, interview).

**FIGURE 1:**
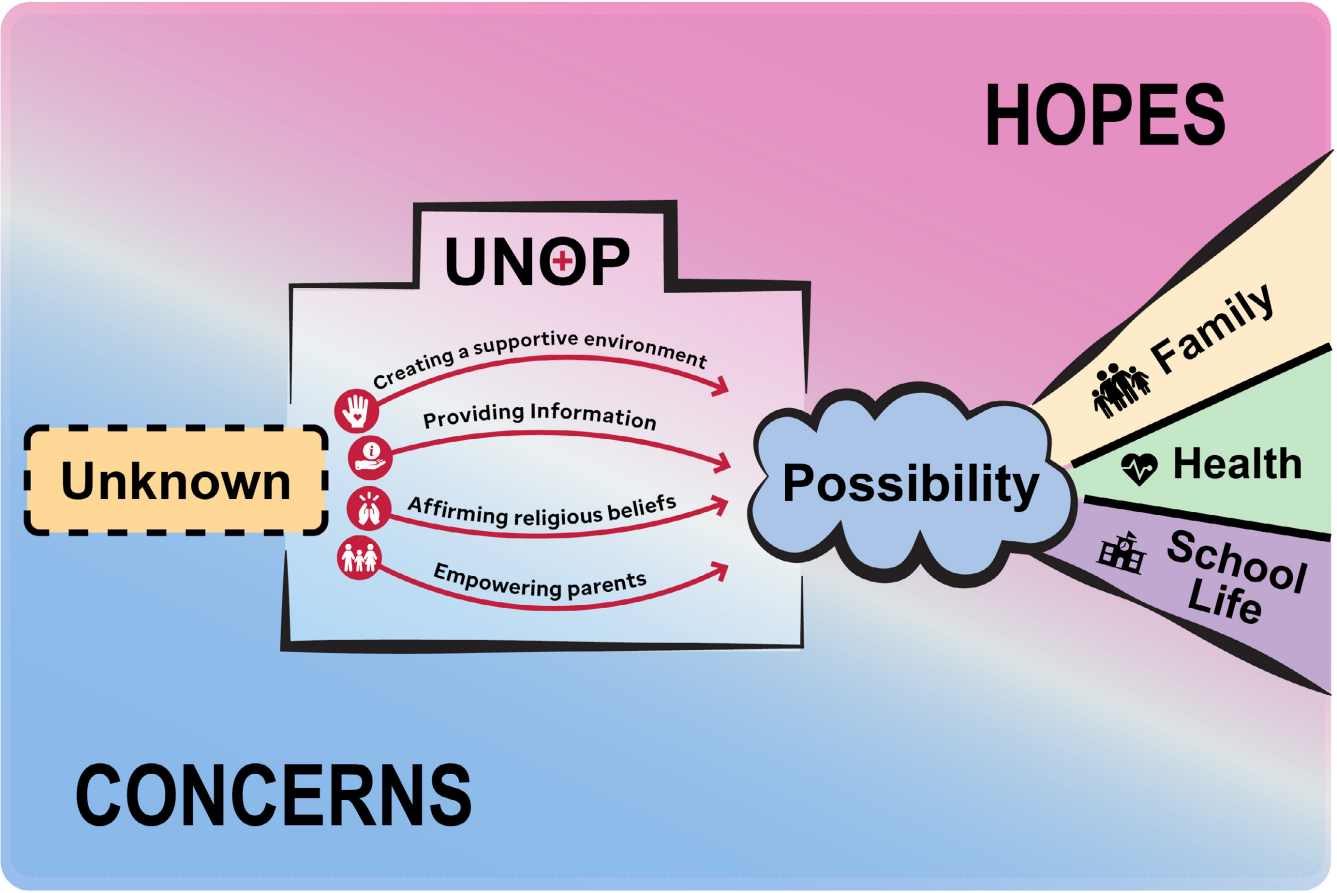
The trajectory of parental hope at diagnosis.

Table 3 includes quotes from parents that further highlight the impact of these four clinical actions on hope. Clinicians at UNOP used these strategies to help parents shift from uncertainty and concern to possibility, control, and ultimately hope for life beyond cancer; Figure 1 depicts this transition.

**TABLE 3:** Discrete ways clinicians support hope.

Creating a supportive environment	“That's a process, I always tell her, trust God first, the doctors and the whole team here, we are in the same page here and they are here to help you.” (mother of a teenager with a hematological malignancy, interview) “I've seen the change in these days of treatment, she has been treated nicely, people here are very kind, I like the way they give her the medicine, with a lot of love and patient, because some children require more patience than others” (mother of a child with a hematological malignancy, interview) “[The team supports us in] what they tell us, the way they encourage us, all that. Nurses are always cheering us up” (father of a teenager with a hematological malignancy, interview)
Providing information	“The important thing is…that you are explaining that there's treatment, a cure.” (father of a child with a hematological malignancy, diagnostic conversation) “What makes me feel good is what you explained to us about the cells she has…” (mother of a teenager with a hematological malignancy, diagnostic conversation) “They also told me that there is hope because 8 out of 10 patients respond very well to chemotherapy.” (father of a teenager with a hematological malignancy, interview)
Affirming religious beliefs	“Also, there are people helping us, they give us spiritual support, which is very important. I feel strengthened, thank God.” (mother of a child with a hematological malignancy, interview) “And also pray for you, for the effort, the knowledge, the intelligence, I think God also gives the doctor his wisdom. I thank him because we have found the illness.” (father of a teenager with a hematological malignancy, diagnostic conversation) “I believe God will do the deed. I hope he gives her a chance to live and give you to chance to treat her.” (father of a child with a hematological malignancy, diagnostic conversation)
Empowering parents	Positive Presence for Child
	“We have to be positive and follow the instructions because you are doing the amazing, and we have to do the rest.” (mother of a child with a hematological malignancy, interview)
	Facilitating Treatment
	“So my advice is never miss the medical appointments.” (father of a teenager with a CNS tumor, interview) “I trust God and the foundation that she is going to be fine, that's why when they asked for donors, they went to donate.” (mother of a teenager with a hematological malignancy, interview)

### 3.4 Hope and parental adjustment

Parents referenced how their hopes were supported by clinical teams as they discussed acceptance, strength, happiness, and peace after diagnosis. Table 4 highlights parental perceptions of emotional benefits.

**TABLE 4:** Parental perceptions of hope's emotional benefits.

Acceptance	“[Before UNOP] I did not want to believe it was happening to him, I said to myself maybe he got hit with something.” (mother of a child with a hematological malignancy, psychosocial intake)
	“But then they gave us the news…it's hard to accept it, it takes days, you have to breathe.” (father of a child with a hematological malignancy, interview)
Strength	“I feel strengthened, thank God.” (mother of a child with a hematological malignancy, interview)
	“Now he is here but he is recovering, and it motivates me.” (father of a child with a hematological malignancy, interview)
	“About my concerns I feel more relaxed now, the first day was hard but these days I feel stronger.” (mother of a child with a hematological malignancy, interview)
Happiness	“I would say that the hopes weight more, are better…yes, that's my incentive and I feel better!” (father of a teenager with a CNS tumor, interview)
	“Once I started seeing changes, I got really happy.” (father of a teenager with a hematological malignancy, interview)
	“She did it in a very nice way that was easy for me to understand, you know how to do your job so we can feel less sad or lonely, because sometimes we feel alone and feel like nobody can help us.” (mother of a child with a hematological malignancy, interview)
Peace	“Now, I'm calmer, doctors have told me that they have not found more cancer cells and that makes me feel better. I still have faith that the illness will never come back.” (mother of a child with a hematological malignancy, interview)
	“One thing that comforts me is that God is the one who puts us in the right place and gave the medicine, if he did that then he will help us get out of this. (mother of a teenager with a hematological malignancy, psychosocial intake)

Initially, many Guatemalan parents grappled with their child's diagnosis wondering what caused their child to become sick. One participant questioned, “I've given her good food, I've taken good care of her, then why is this happening?” (mother of a child with a hematological malignancy, psychosocial intake). As parents discussed finding acceptance, they often mentioned hope, “But like she said, nothing is impossible. With treatment they try to cure the children. Whatever happens we must accept the situation” (mother of a child with a hematological malignancy, interview).

Throughout diagnostic conversations and interviews, parents discussed how support, religion, and hope contributed to new‐found strength. One mother explained, “When you come here, you arrive petrified but here you recover your strength. When you started to talk to her and she started to analyze, she told me mom this place is nice and they are very kind. I said yes, they are lovely and you will be fine” (mother of a teenager with a hematological malignancy, interview). Another mother reflected on the strength she derived from religion, “In the middle of all this I try to give up and say God I know you know the things happening…I know he'll be okay; I want to think that way” (mother of a child with a hematological malignancy, diagnostic conversation).

Many parents described happiness. One father said, “The important thing is…that you are explaining that there's treatment, a cure. That makes me feel a little bit happy because there's still hope” (father of a child with a hematological malignancy, diagnostic conversation). Participants also associated peace with hope. One commented, “yes, we have a lot of concerns, but we close our eyes and pray, and we feel relieved. I teach him to do it. There is always hope” (mother of a child with a hematological malignancy, interview).

Finally, parents explicitly discussed how UNOPs supportive environment, knowledge, and empowerment helped alleviate concerns and allowed them to care for their own needs. One parent described time before UNOP saying, “…during those 4 days I couldn't sleep any because I was too scared and uncomfortable,” later commenting, “I feel I can rest here, although my distress is always there, I can rest here,” (mother of a child with a solid tumor, interview). Another participant responded to a psychologist's comment about hope explaining, “We were feeling so weak, and yesterday we were able to go, have lunch, and we ate in peace” (mother of a child with a hematological malignancy, diagnostic conversation), highlighting the impact of hope on self‐care.

## 4 DISCUSSION

From the time of diagnosis, Guatemalan parents expressed hopes and concerns pertaining to the entire cancer continuum and described how support from the UNOP clinical team helped them shift their focus from concern toward hope. Ultimately, parents associated hope with acceptance, strength, happiness, peace, and coping. These findings are aligned with studies examining hope among parents of pediatric oncology patients in HICs.^1^, ^4^ Our results further establish the relevance of hope in LMICs at the time of diagnosis for all populations, including those treated with curative intent.

Supporting hope has been previously defined as one of eight communication functions essential to pediatric oncology.^2^ While this function was initially established through work with populations within the United States, our results confirm its significance within Guatemala. Furthermore, our results identify four discrete actions that parents cited during discussions of hope, *creating a supportive environment*, *providing information*, *affirming religious beliefs*, *and empowering parents*, which clinicians can use to operationalize this function regardless of the cultural context. Table 5 highlights cultural factors including literacy, religious beliefs, and cultural norms that may impact the role of the clinician in supporting hope and suggests ways to adapt clinical actions accordingly.

**TABLE 5:** Supporting hope across clinical settings.

*Core clinical actions*	Cultural factors to consider	Possible adaptations
Creating a supportive environment	Family structure Relationship with community	Discuss family's support preferences to understand the types and level of support needed (may involve extended family or community members) Understand impact of family, community, and hospital resources on hope Demonstrate curiosity about the family outside of cancer treatment
Providing information	Family characteristics Medical models Prior experiences with cancer, including potential misconceptions or fatalistic beliefs Role of the child	Consider tailored cancer education that accounts for family's unique lived experiences based on occupation, culture, etc. Utilize an interpreter when possible Include both allopathic descriptions and metaphorical descriptions of cancer Engage in bidirectional information exchange; accept each family's cultural beliefs while guiding conversation to facilitate scientific understanding Consider incorporation of child into communication and care
Affirming religious beliefs	Religion and traditional beliefs about cancer Cultural norms about the discussion of religion in medicine	Maintain curiosity about parents' beliefs or religious affiliations and how they may aid in coping Tailor religion‐related communication to the beliefs of the family and their desire to/not to discuss religion Provide opportunities for community religious leaders to provide support
Empowering parents	Decision‐making models Parental preference for involvement in decision‐making Parental self‐efficacy	Seek to understand cultural hierarchies around medical decision making Encourage shared decision‐making models by empowering parents to make treatment decisions Share ways parents can participate in their child's care and promote parental self‐efficacy
Recurrent diagnostic conversations	Awareness regarding childhood cancer Emotional state of parents at the time of diagnosis Treatment abandonment	Consider ways to have recurrent conversations to ensure proper information exchange and provide opportunities for families to ask questions Utilize multidisciplinary providers to evaluate parental understanding, respond to family emotions, and reinforce information during follow‐up conversations Address factors related to abandonment and importance of treatment adherence
Involvement of multidisciplinary team	Disciplines available at the cancer center Community perceptions regarding members of the medical team	Include multidisciplinary team members (psychologist, social worker, nurse, patient educator, chaplain/spiritual care provider) in case discussions and patient care Consider roles and responsibilities for each team member and clearly delineate

The experiences of Guatemalan parents reflect the goal‐pathway‐agency model of hope theory.^8^ During the pre‐diagnosis period, participants' concerns about the unknown reflected a perceived lack of pathways and agency. Throughout the diagnostic process, clinicians provided information to help parents realize a pathway to survival and empowered them to recover agency, which facilitated feelings of hope. Furthermore, our findings reflect the conclusion that the locus of hope is dependent on cultural setting.^7^ Parents in Guatemala frequently mentioned religion and cited the power of supernatural beings as integral to hope, suggesting an external locus. By affirming religious beliefs, Guatemalan clinicians reinforced parents' external anchors of hope and utilized them to build trust, strengthen faith in the medical team, and emphasize possibility. The supportive environment at UNOP allows parents to expand their anchors of hope to include the medical team, thereby supporting the process by which parents find hope.

Finally, our results demonstrate how supporting hope may influence outcomes. This study's findings suggest that hope may have a role in facilitating parental acceptance and coping following diagnosis. As clinicians supported hope, they empowered parents, emphasized dignity, and demonstrated respect, interactions which may be associated with longer‐term outcomes including increased quality of care and lower rates of treatment abandonment.^18^ Perhaps the most telling evidence of the importance of culturally sensitive hope communication on outcomes is the resounding success of UNOPs implementation of integrated psychosocial services through *medicina integral*. This multidisciplinary approach included tailored services and adapted clinical conversations that reflected the cultural needs of the Guatemalan people and facilitated clinicians' ability to support hope and build a therapeutic alliance. Implementation of the *medicina integral* team contributed to treatment abandonment rates falling from 27% to 7%.^19^

This study had several limitations. Understanding hope was not the primary objective of the original study and thus was not central to the design of the interview guide. Data was exclusively collected during the diagnostic period; future work is needed to assess how hope evolves throughout treatment. In addition, this analysis focused on hope at UNOP, a single public‐private hospital in Guatemala, a predominantly Christian, upper‐middle income country. Further work should explore parental hope in other contexts to understand the applicability of identified themes in regions with different cultures and resources. While bilingual study team members checked translations, English‐Spanish translation during study tool development, data collection, and analysis may have impacted results. The use of Spanish during data collection may have limited the ability of parents whose primary language was a Mayan dialect to fully express themselves. Additionally, future work is needed to explore pediatric patients' perspectives on hope as this study included only parent participants.

## 5 CONCLUSION

Our results highlight the relevance of parental hope to pediatric oncology regardless of cultural setting. While the importance of hope appears to be universal, culture affects how it manifests, impacting parents' hope‐related needs and influencing the role of the clinician in supporting hope. These results indicate that clinicians, regardless of resource setting, should tailor communication based on families' cultural contexts. While previous studies identified processes that underly the ways clinicians support hope, this study delineated four discrete actions that facilitate these processes providing insight into how hope can be integrated into clinical communication. Further work should explore similar themes in other contexts to expand on the role of culture in hope and focus on interventions that promote culturally adaptive hope communication.

## AUTHOR CONTRIBUTIONS

**Anneliese H. Williams:** Formal analysis (lead); visualization (equal); writing – original draft (lead); writing – review and editing (equal). **Silvia Rivas:** Methodology (supporting); project administration (equal); resources (equal); writing – review and editing (equal). **Lucia Fuentes:** Investigation (lead); methodology (supporting); resources (equal); writing – review and editing (equal). **Ana Caceres‐Serrano:** Investigation (lead); methodology (supporting); resources (equal); writing – review and editing (equal). **Gia Ferrara:** Formal analysis (supporting); writing – review and editing (equal). **Tegan Reeves:** Formal analysis (supporting); writing – review and editing (equal). **Federico Antillon‐Klussmann:** Methodology (supporting); project administration (equal); resources (equal); writing – review and editing (equal). **Carlos Rodriguez‐Galindo:** Methodology (supporting); writing – review and editing (equal). **Jennifer W. Mack:** Methodology (supporting); writing – review and editing (equal). **Dylan E. Graetz:** Conceptualization (lead); formal analysis (supporting); investigation (supporting); methodology (lead); project administration (equal); supervision (equal); visualization (equal); writing – review and editing (equal).

## FUNDING INFORMATION

This work was funded by American Lebanese Syrian Associated Charities of St. Jude Children's Research Hospital and a Conquer Cancer Young Investigator Award (award number 17290). Any opinions, findings, and conclusions expressed in this material are those of the authors and do not necessarily reflect those of the American Society of Clinical Oncology or Conquer Cancer.

## CONFLICT OF INTEREST STATEMENT

The authors have no conflicts of interest relevant to this article to disclose.

## Data Availability

The data that support the findings of this study are available from the corresponding author upon reasonable request.
